# Barriers to initiating tuberculosis treatment in sub-Saharan Africa: a systematic review focused on children and youth

**DOI:** 10.1080/16549716.2017.1290317

**Published:** 2017-06-09

**Authors:** Brittney J. Sullivan, B. Emily Esmaili, Coleen K. Cunningham

**Affiliations:** ^a^ School of Nursing, Duke University, Durham, NC, USA; ^b^ Duke Global Health Institute; ^c^ Department of Science and Society, Duke University, Durham, NC, USA; ^d^ School of Medicine, Duke University, Durham, NC, USA

**Keywords:** Access, delay, global health, health systems, pediatrics

## Abstract

**Background**: Tuberculosis (TB) is the deadliest infectious disease globally, with 10.4 million people infected and more than 1.8 million deaths in 2015. TB is a preventable, treatable, and curable disease, yet there are numerous barriers to initiating treatment. These barriers to treatment are exacerbated in low-resource settings and may be compounded by factors related to childhood.

**Objective**: Timely initiation of tuberculosis (TB) treatment is critical to reducing disease transmission and improving patient outcomes. The aim of this paper is to describe patient- and system-level barriers to TB treatment initiation specifically for children and youth in sub-Saharan Africa through systematic review of the literature.

**Design**: This review was conducted in October 2015 in accordance with preferred reporting items for systematic reviews and meta-analyses (PRISMA) guidelines. Six databases were searched to identify studies where primary or secondary objectives were related to barriers to TB treatment initiation and which included children or youth 0–24 years of age.

**Results**: A total of 1490 manuscripts met screening criteria; 152 met criteria for full-text review and 47 for analysis. Patient-level barriers included limited knowledge, attitudes and beliefs regarding TB, and economic burdens. System-level barriers included centralization of services, health system delays, and geographical access to healthcare. Of the 47 studies included, 7 evaluated cost, 19 health-seeking behaviors, and 29 health system infrastructure. Only 4 studies primarily assessed pediatric cohorts yet all 47 studies were inclusive of children.

**Conclusions**: Recognizing and removing barriers to treatment initiation for pediatric TB in sub-Saharan Africa are critical. Both patient- and system-level barriers must be better researched in order to improve patient outcomes.

## Background

*Mycobacterium tuberculosis* (TB) is the leading infectious cause of death worldwide, surpassing HIV/A.I.D.S [1]. In 2015, TB killed 1.8 million people with 95% of cases and deaths in developing countries [1]. TB is an airborne infectious agent requiring at minimum an intensive six-month medication regimen for bacteriologic cure [1]. Timely initiation and correct treatment of TB are critical to reduce disease transmission and improve patient outcomes. However, barriers to treatment initiation exist at both the patient and system levels. Patient-level barriers such as perception of illness, stigma, knowledge about TB, delay in seeking care and initiating treatment, and direct and indirect costs all cause delayed treatment [2,3]. Health system barriers include resource capacity such as the availability of laboratory tests, accessibility of different levels of care, and costs. Patient costs associated with TB treatment often cause patients and families to fall into a ‘medical poverty trap’ [4,5].

Pulmonary TB outcomes in children are favorable when treated; however, data are limited regarding outcomes for children (0–18 years) and youth (15–24 years) [6–10]. This research gap is in part due to lack of standardized definitions of age cohorts (i.e. pediatric, child, adolescent, youth) as well as lack of political and community commitment to this age group [11]. Barriers to involving youth in research, coupled with developmental transitions, additional responsibilities associated with education and employment, and dependence on family commitment, may cause youth to be understudied [12].

Pediatric cases (0–18 years) account for 10% of all new and relapse cases of TB in the African region, as compared to 6.5% globally [1]. Additionally, the African region has the highest rate of TB in children and youth compared to any other region [1]. Despite the high burden of disease among younger age groups, barriers are most often studied in adult populations. As a result, barriers to treatment initiation in children and youth are less well understood [12,13]. The objective of this review is to determine patient- and system-level barriers to treatment initiation in sub-Saharan Africa (SSA) with an emphasis on children and youth diagnosed with TB, through systematic review of the literature.

## Methods

### Search strategy

We systematically searched six health databases for literature pertaining to pediatric and youth TB in SSA. Specified terms were agreed upon by the authors and adapted to each database. The protocol is provided in Supplement 1 and full search criteria for each database are provided in Supplement 2.

### Selection criteria

**Figure 1.**
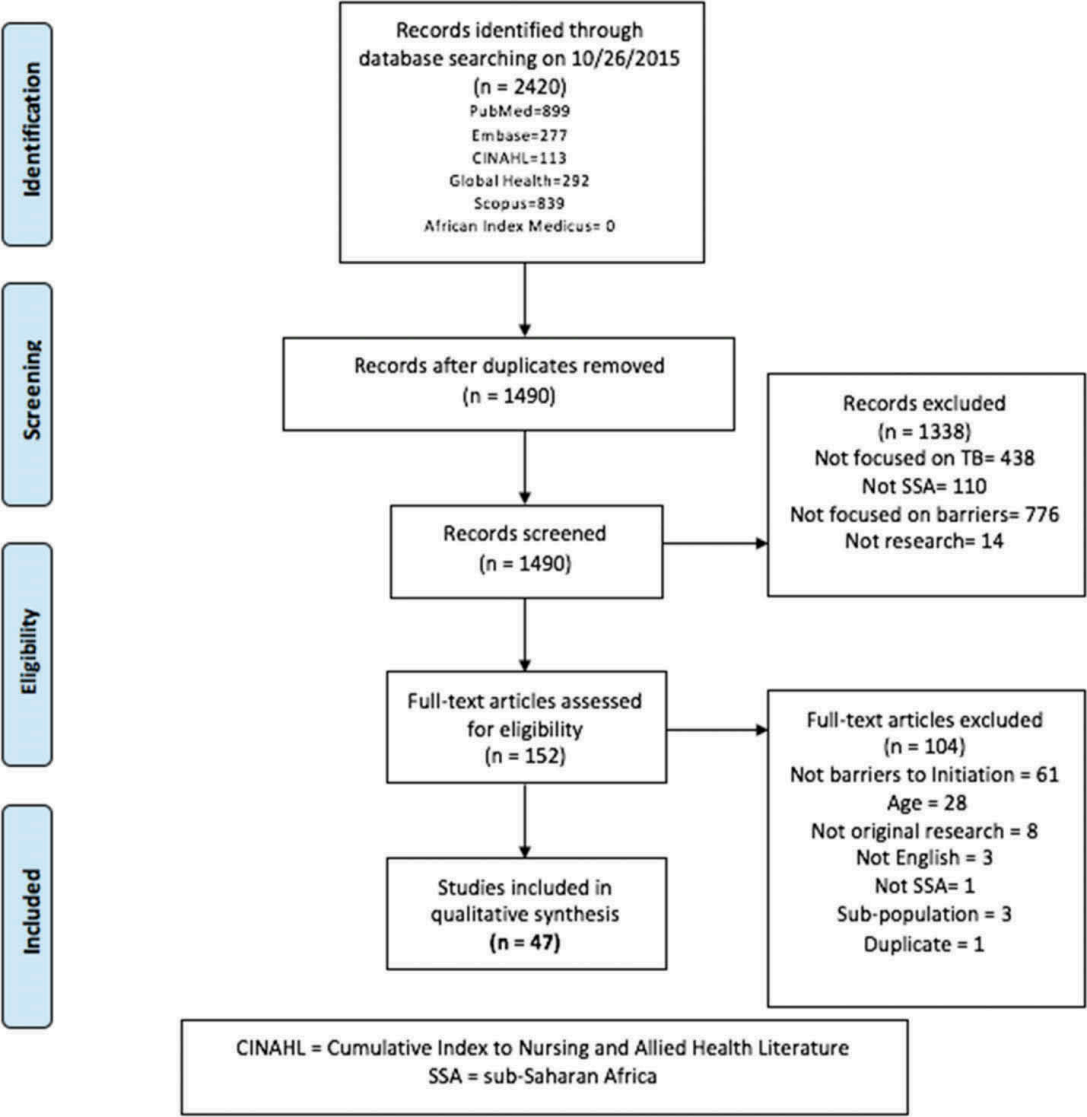
P.R.I.S.M.A flow diagram.

Inclusion criteria were established by the authors a priori based upon preliminary literature searches. Preferred reporting items for systematic reviews and meta-analyses (PRISMA) guidelines were followed for inclusion and exclusion (Figure 1). Articles were included through 26 October 2015. All potentially eligible original research studies with abstracts in English were reviewed. Inclusion criteria were: a primary or secondary aim of the study addressing barriers to TB treatment initiation for children or youth in SSA. Definitions of barriers, age groups, treatment initiation, and other variables are provided in Table 1. All publication dates and all study designs were included.

**Table 1.** Definitions used in systematic review.

Variable	Definition
Barrier	Obstacle preventing TB treatment initiation and treatment access. Includes: cost, infrastructure, and health-seeking behaviors.
Treatment initiation	TB medication start date.
Child (pediatric)	0–18 (UNICEF)[14]
Adolescent	10–19 years (UNICEF, WHO, UNFPA)[14]
Youth	15–24 (WHO, UN, UNESCO, UNICEF)[14]
Patient-level (P)	Individual-level factors and perceptions. Includes patient costs, health-seeking behavior, and internal/external stigma.
System-level (S)	Characteristics of health systems. Includes health system delays, laboratory capacity, geography, health system infrastructure beyond the individual (i.e. information technology systems), and provider attitudes towards TB.
Cost	Direct or indirect economic burden to family, guardian, and/or patient associated with TB care. *Part of the cost was incurred *prior to* treatment initiation, i.e. costs incurred while obtaining diagnosis or between diagnosis and treatment were included in analysis.
Direct cost	Out-of-pocket expenses for transportation, food, medicine, etc.
Indirect cost	Lost wages due to time spent seeking care or inability to work.
Catastrophic medical expenses	When a household’s total out-of-pocket health payments are equal to or exceed 40% of the household’s capacity to pay [15].
Health-seeking behavior	Navigation of the health system. Includes care pathways of providers (formal and informal healthcare providers, private and public sector, traditional healers, health extension workers, herbalists, nurses, physicians, etc.) sought prior to appropriate TB diagnosis and treatment. Also includes knowledge, attitudes, and beliefs regarding TB.
Infrastructure	The geography and access to care, laboratory capacity, level of care and health policies (centralized versus decentralized care), health system errors (initial default), and quality of health services delivery.
Initial default	If a patient is diagnosed with TB but does not initiate treatment.

### Data extraction and analysis

All search results were entered into EndNote X7 (Thomson Reuters Scientific Inc., Carlsbad, CA, USA) and duplicates were removed. Two authors (BJS and BEE) reviewed titles and abstracts with the aim of removing publications that were unlikely to meet the inclusion criteria. After both authors reviewed 20% of articles and demonstrated greater than 95% inter-rater reliability, the remaining articles were assessed independently. Discrepancies were resolved through discussion with all three authors until agreement was reached.

Garrard’s matrix strategy for abstracting was used to build a Microsoft *Excel* spreadsheet and abstract full texts of the remaining articles [16]. The following data were extracted from the included articles: author, publication year, title, journal, country and setting, study aim and design, outcomes and barriers measured, sample size, covariates, strengths and limitations of each study, 95% confidence intervals and effect sizes (if reported), if HIV was measured as a variable, and if pediatric and youth cases were specifically described in the findings or discussion.

## Results

### Study selection

After removing duplicates, a total of 1490 articles were included. There were 1338 articles excluded and 47 articles were retained for full-text review (Figure 1). The 47 studies were from 14 countries with 16 studies conducted in South Africa (Table 2). While all 47 studies included children or youth in their sample, only 4 studies were pediatric-only cohorts [17–20]. Barriers were identified as either patient- or system-level (or both), and whether the barrier related to (1) cost, (2) infrastructure, and/or (3) health-seeking behavior (Table 2).

**Table 2.** Included articles.

Citation	Author	Year	Title/journal	Country	Type of barrier and level of analysis	Pediatric specific
[17]	Beyers, N., R. P. Gie, H. S. Schaaf, S. van Zyl, E. D. Nel, J. M. Talent and P. R. Donald	1994	Delay in the diagnosis, notification and initiation of treatment and compliance in children with tuberculosis. Tuber Lung Dis 75(4): 260–265	South Africa	Infrastructure (P + S)	Yes
[21]	Salaniponi, F. M., A. D. Harries, H. T. Banda, C. Kang’ombe, N. Mphasa, A. Mwale, B. Upindi, T. E. Nyirenda, A. Banerjee and M. J. Boeree	2000	Care seeking behaviour and diagnostic processes in patients with smear-positive pulmonary tuberculosis in Malawi. Int J Tuberc Lung Dis 4(4): 327–332	Malawi	Health-seeking behavior (P)	No
[22]	Lienhardt, C., J. Rowley, K. Manneh, G. Lahai, D. Needham, P. Milligan and K. P. McAdam	2001	Factors affecting time delay to treatment in a tuberculosis control programme in a sub-Saharan African country: the experience of The Gambia. Int J Tuberc Lung Dis 5(3): 233–239	Gambia	Infrastructure (P + S)	No
[23]	Edginton, M. E., C. S. Sekatane and S. J. Goldstein	2002	Patients’ beliefs: do they affect tuberculosis control? A study in a rural district of South Africa. Int J Tuberc Lung Dis 6(12): 1075–1082	South Africa	Health-seeking behavior (P)	No
[24]	Enwuru, C. A., E. O. Idigbe, N. V. Ezeobi and A. F. Otegbeye	2002	Care-seeking behavioural patterns, awareness and diagnostic processes in patients with smear- and culture-positive pulmonary tuberculosis in Lagos, Nigeria. Trans R Soc Trop Med Hyg 96(6): 614–616	Nigeria	Health-seeking behavior (P)	No
[25]	Eastwood, S. V. and P. C. Hill	2004	A gender-focused qualitative study of barriers to accessing tuberculosis treatment in The Gambia, West Africa. Int J Tuberc Lung Dis 8(1): 70–75	Gambia	Health-seeking behavior (P + S)	No
[26]	Martin, A., J. P. Baptiste and G. Krieger	2004	Respiratory infections: SARS and tuberculosis. Clinics in Occupational and Environmental Medicine 4(1): 189–204	Chad	Health-seeking behavior (P), infrastructure (S)	No
[27]	Cambanis, A., M. A. Yassin, A. Ramsay, S. Bertel Squire, I. Arbide and L. E. Cuevas	2005	Rural poverty and delayed presentation to tuberculosis services in Ethiopia. Trop Med Int Health 10(4): 330–335	Ethiopia	Health-seeking behavior (P)	No
[28]	Edginton, M. E., M. L. Wong, R. Phofa, D. Mahlaba and H. J. Hodkinson	2005	Tuberculosis at Chris Hani Baragwanath Hospital: numbers of patients diagnosed and outcomes of referrals to district clinics. Int J Tuberc Lung Dis 9(4): 398–402	South Africa	Health-seeking behavior (P + S), infrastructure (S)	No
[29]	Yimer, S., G. Bjune and G. Alene	2005	Diagnostic and treatment delay among pulmonary tuberculosis patients in Ethiopia: a cross sectional study. BMC Infect Dis 5: 112	Ethiopia	Health-seeking behavior (P + S), infrastructure (P + S)	No
[30]	Barker, R. D., F. J. C. Millard, J. Malatsi, L. Mkoana, T. Ngoatwana, S. Agarawal and S. De Valliere	2006	Traditional healers, treatment delay, performance status and death from TB in rural South Africa. International Journal of Tuberculosis and Lung Disease 10(6): 670–675	South Africa	Health-seeking behavior (P + S)	No
[31]	Dembele, S. M., H. Z. Ouedraogo, A. I. Combary, B. Sondo, J. Macq and B. Dujardin	2006	Are patients who present spontaneously with PTB symptoms to the health services in Burkina Faso well managed? Int J Tuberc Lung Dis 10(4): 436–440	Burkina Faso	Infrastructure (S)	No
[18]	Engelbrecht, A. L., B. J. Marais, P. R. Donald and H. S. Schaaf	2006	A critical look at the diagnostic value of culture-confirmation in childhood tuberculosis. J Infect 53(6): 364–369	South Africa	Infrastructure (S)	Yes
[32]	Botha, E., S. den Boon, K. A. Lawrence, H. Reuter, S. Verver, C. J. Lombard, C. Dye, D. A. Enarson and N. Beyers	2008	From suspect to patient: tuberculosis diagnosis and treatment initiation in health facilities in South Africa. Int J Tuberc Lung Dis 12(8): 936–941	South Africa	Infrastructure (S)	No
[33]	Botha, E., S. Den Boon, S. Verver, R. Dunbar, K. A. Lawrence, M. Bosman, D. A. Enarson, I. Toms and N. Beyers	2008	Initial default from tuberculosis treatment: how often does it happen and what are the reasons? Int J Tuberc Lung Dis 12(7): 820–823	South Africa	Infrastructure (S)	No
[34]	den Boon, S., S. Verver, C. J. Lombard, E. D. Bateman, E. M. Irusen, D. A. Enarson, M. W. Borgdorff and N. Beyers	2008	Comparison of symptoms and treatment outcomes between actively and passively detected tuberculosis cases: the additional value of active case finding. Epidemiol Infect 136(10): 1342–1349	South Africa	Infrastructure (S)	No
[35]	Mfinanga, S. G., B. K. Mutayoba, A. Kahwa, G. Kimaro, R. Mtandu, E. Ngadaya, S. Egwaga and A. Y. Kitua	2008	The magnitude and factors associated with delays in management of smear positive tuberculosis in Dar es Salaam, Tanzania. BMC Health Serv Res 8: 158	Tanzania	Health-seeking behavior (P + S)	No
[36]	Dodor, E. A., S. Kelly and K. Neal	2009	Health professionals as stigmatisers of tuberculosis: insights from community members and patients with TB in an urban district in Ghana. Psychol Health Med 14(3): 301–310	Ghana	Health-seeking behavior (P + S)	No
[37]	Datiko, D. G. and B. Lindtjørn	2010	Cost and cost-effectiveness of treating smear-positive tuberculosis by health extension workers in Ethiopia: an ancillary cost-effectiveness analysis of community randomized trial. PLoS ONE 5(2)	Ethiopia	Cost (P + S), infrastructure (S)	No
[38]	Yimer, S., C. Holm-Hansen, T. Yimaldu and G. Bjune	2009	Health care seeking among pulmonary tuberculosis suspects and patients in rural Ethiopia: a community-based study. BMC Public Health 9: 454	Ethiopia	Health-seeking behavior (P + S), infrastructure (P + S)	No
[39]	Sendagire, I., M. Schim Van der Loeff, M. Mubiru, J. Konde-Lule and F. Cobelens	2010	Long delays and missed opportunities in diagnosing smear-positive pulmonary tuberculosis in Kampala, Uganda: a cross-sectional study. PLoS One 5(12): e14459	Uganda	Health-seeking behavior (P + S), infrastructure (P + S)	No
[40]	Vassall, A., A. Seme, P. Compernolle and F. Meheus	2010	Patient costs of accessing collaborative tuberculosis and human immunodeficiency virus interventions in Ethiopia. Int J Tuberc Lung Dis 14(5): 604–610	Ethiopia	Cost (P)	No
[41]	Mauch, V., N. Woods, B. Kirubi, H. Kipruto, J. Sitienei and E. Klinkenberg	2011	Assessing access barriers to tuberculosis care with the tool to Estimate Patients’ Costs: pilot results from two districts in Kenya. BMC Public Health 11: 43	Kenya	Cost (P)	No
[42]	Dodor, E. A.	2012	The feelings and experiences of patients with tuberculosis in the Sekondi-Takoradi Metropolitan district: implications for TB control efforts. Ghana Med J 46(4): 211–218	Ghana	Health-seeking behavior (P)	No
[43]	Ngangro, N. N., D. Ngarhounoum, M. N. Ngangro, N. Rangar, M. G. Siriwardana, V. H. des Fontaines and P. Chauvin	2012	Pulmonary tuberculosis diagnostic delays in Chad: a multicenter, hospital-based survey in Ndjamena and Moundou. BMC Public Health 12: 513	Chad	Infrastructure (P + S), health-seeking behavior (P + S)	No
[44]	Scott, V., V. Azevedo and J. Caldwell	2012	Improving access and quality of care in a TB control programme. SAMJ – South African Medical Journal 102(11): 837–840	South Africa	Infrastructure (S)	No
[19]	Seddon, J. A., A. C. Hesseling, M. Willemse, P. R. Donald and H. S. Schaaf	2012	Culture-confirmed multidrug-resistant tuberculosis in children: clinical features, treatment, and outcome. Clin Infect Dis 54(2): 157–166	South Africa	Infrastructure (P + S)	Yes
[45]	Umar, N. A., I. Abubakar, R. Fordham and M. Bachmann	2012	Direct costs of pulmonary tuberculosis among patients receiving treatment in Bauchi State, Nigeria. Int J Tuberc Lung Dis 16(6): 835–840	Nigeria	Cost (P)	No
[46]	Cowan, J., J. G. Cowan, S. Barnhart, S. Demamu, D. Fiseha, W. Graham, E. Melese, L. Reason, F. T. Asfaw, G. Feleke and B. Feleke	2013	A qualitative assessment of challenges to tuberculosis management and prevention in Northern Ethiopia. International Journal of Tuberculosis and Lung Disease 17(8): 1071–1075	Ethiopia	Infrastructure (S)	No
[20]	Zimri, K., A. C. Hesseling, P. Godfrey-Faussett, H. S. Schaaf and J. A. Seddon	2012	Why do child contacts of multidrug-resistant tuberculosis not come to the assessment clinic? Public Health Action 2(3): 71–75	South Africa	Health-seeking behavior (P)	Yes
[47]	Ebonwu, J. I., K. S. Tint and C. Ihekweazu	2013	Low treatment initiation rates among multidrug-resistant tuberculosis patients in Gauteng, South Africa, 2011. Int J Tuberc Lung Dis 17(8): 1043–1048	South Africa	Infrastructure (P + S)	No
[48]	Jacobson, K. R., D. Theron, E. A. Kendall, M. F. Franke, M. Barnard, P. D. van Helden, T. C. Victor, E. M. Streicher, M. B. Murray and R. M. Warren	2013	Implementation of genotype MTBDRplus reduces time to multidrug-resistant tuberculosis therapy initiation in South Africa. Clin Infect Dis 56(4): 503–508	South Africa	Infrastructure (S)	No
[49]	Yassin, M. A., D. G. Datiko, O. Tulloch, P. Markos, M. Aschalew, E. B. Shargie, M. H. Dangisso, R. Komatsu, S. Sahu, L. Blok, L. E. Cuevas and S. Theobald	2013	Innovative community-based approaches doubled tuberculosis case notification and improve treatment outcome in Southern Ethiopia. PLoS One 8(5): e63174	Ethiopia	Infrastructure (S)	No
[50]	Ansa, G. A., J. D. Walley, K. Siddiqi and X. Wei	2014	Delivering TB/HIV services in Ghana: a comparative study of service delivery models. Trans R Soc Trop Med Hyg 108(9): 560–567	Ghana	Infrastructure (S)	No
[51]	Asefa, A. and W. Teshome	2014	Total delay in treatment among smear positive pulmonary tuberculosis patients in five primary health centers, Southern Ethiopia: A cross sectional study. PLoS ONE 9(7): e102884.	Ethiopia	Health-seeking behavior (P + S), infrastructure (P + S)	No
[52]	Biya, O., S. Gidado, A. Abraham, N. Waziri, P. Nguku, P. Nsubuga, I. Suleman, A. Oyemakinde, A. Nasidi and K. Sabitu	2014	Knowledge, care-seeking behavior, and factors associated with patient delay among newly-diagnosed pulmonary tuberculosis patients, Federal Capital Territory, Nigeria, 2010. Pan Afr Med J 18 Suppl 1: 6	Nigeria	Health-seeking behavior (P)	No
[53]	Dlamini-Mvelase, N. R., L. Werner, R. Phili, L. P. Cele and K. P. Mlisana	2014	Effects of introducing Xpert MTB/RIF test on multi-drug resistant tuberculosis diagnosis in KwaZulu-Natal South Africa. BMC Infect Dis 14: 442	South Africa	Infrasturcture (S)	No
[54]	Laokri, S., M. Dramaix-Wilmet, F. Kassa, S. Anagonou and B. Dujardin	2014	Assessing the economic burden of illness for tuberculosis patients in Benin: determinants and consequences of catastrophic health expenditures and inequities. Trop Med Int Health 19(10): 1249–1258	Benin	Cost (P)	No
[55]	Makwakwa, L., M. L. Sheu, C. Y. Chiang, S. L. Lin and P. W. Chang	2014	Patient and health system delays in the diagnosis and treatment of new and retreatment pulmonary tuberculosis cases in Malawi. BMC Infect Dis 14: 132	Malawi	Health-seeking behavior (P), infrastructure (S)	No
[56]	Virenfeldt, J., F. Rudolf, C. Camara, A. Furtado, V. Gomes, P. Aaby, E. Petersen and C. Wejse	2014	Treatment delay affects clinical severity of tuberculosis: a longitudinal cohort study. BMJ Open 4(6): e004818	Guinea-Bissau	Infrastructure (P + S)	No
[57]	Yimer, S. A., G. A. Bjune and C. Holm-Hansen	2014	Time to first consultation, diagnosis and treatment of TB among patients attending a referral hospital in Northwest, Ethiopia. BMC Infectious Diseases 14(1): 19–19 11p	Ethiopia	Infrastructure (P + S)	No
[58]	Yitayal, M., A. Aseffa, G. Andargie, L. Wassie and M. Abebe	2014	Assessment of cost of tuberculosis to patients and their families: a cross-sectional study at Addet Health Center, Yilmana Densa District, Amhara National Regional State. Ethiop Med J Suppl 1: 23–30	Ethiopia	Cost (P)	No
[59]	Abimbola, S., K. N. Ukwaja, C. C. Onyedum, J. Negin, S. Jan and A. L. C. Martiniuk	2015	Transaction costs of access to health care: implications of the care-seeking pathways of tuberculosis patients for health system governance in Nigeria. Global Public Health 10(9): 1060–1077	Nigeria	Cost (P), health-seeking behavior (P)	No
[60]	Cox, H. S., J. F. Daniels, O. Muller, M. P. Nicol, V. Cox, G. van Cutsem, S. Moyo, V. De Azevedo and J. Hughes	2015	Impact of decentralized care and the Xpert MTB/RIF test on rifampicin-resistant tuberculosis treatment initiation in Khayelitsha, South Africa. Open Forum Infectious Diseases 2(1): ofv014.	South Africa	Infrastructure (S)	No
[61]	Cremers, A. L., M. M. de Laat, N. Kapata, R. Gerrets, K. Klipstein-Grobusch and M. P. Grobusch	2015	Assessing the consequences of stigma for tuberculosis patients in urban Zambia. PLoS One 10(3): e0119861	Zambia	Health-seeking behavior (P + S)	No
[62]	Ross, J. M., A. Cattamanchi, C. R. Miller, A. J. Tatem, A. Katamba, P. Haguma, M. A. Handley and J. L. Davis	2015	Investigating barriers to tuberculosis evaluation in Uganda using geographic information systems. American Journal of Tropical Medicine and Hygiene 93(4): 733–738	Uganda	Infrastructure (S)	No
[63]	Van Den Handel, T., K. H. Hampton, I. Sanne, W. Stevens, R. Crous and A. Van Rie	2015	The impact of Xpert® MTB/RIF in sparsely populated rural settings. International Journal of Tuberculosis and Lung Disease 19(4): 392–398	South Africa	Infrastructure (S)	No

Note: Barriers: cost, infrastructure, health-seeking behavior. Level of analysis: patient, system.

### Cost as a barrier to TB treatment initiation

Seven articles identified cost as a barrier to TB treatment initiation. Costs included: direct, indirect, system, or caregiver costs; as well as costs incurred prior to diagnosis and between diagnosis and treatment initiation (Supplement 3) [37,40,41,45,54,58,59]. All articles identifying costs cited patient-level costs as a barrier, while only one article considered cost as a barrier from the health system perspective [37]. All studies described direct out-of-pocket costs for patients. Five studies described indirect costs [37,40,41,54,58]. One study reported the median total of direct and indirect costs was equivalent to 45% of median annual individual incomes ($350 U.S.D) with indirect costs accounting for 85% of total costs [41].

Two articles described costs associated with caretakers in which the cost burden to guardians or caretakers was high [40,41]. In addition, one study also measured intangible costs (non-monetary costs affecting quality of life, such as pain, suffering, and social stigma) in addition to catastrophic expenditures [54]. Another study measured transaction costs, the difference between total direct costs incurred by a patient and total direct costs incurred prior to the first contact with a National TB Control Program provider [59]. In one of the only intervention studies, Abimbola et al. (2015) cited that interventions for reducing transaction costs should include effective decentralization of services to integrate TB care with primary healthcare. Studies suggested that encouraging the engagement of communities to help address health education and facilitation of referral linkages among formal and informal care providers may reduce costs. None of the cost studies specifically addressed pediatric or youth barriers beyond those of the general population.

### Infrastructure as a barrier to TB treatment initiation

Infrastructure as a barrier to TB treatment was included in 29 articles (Supplement 5) [17–19,22,26–29,31–34,37,39,43,44,46–51,53,55–57,60,62,63]. Three of the four pediatric-only studies included infrastructure barriers [17–19]. Infrastructure includes structural or organizational issues such as geography or distance to TB treatment centers, laboratory capacity, level of service, health services delay or quality of care, and initial loss to follow-up (formerly known as initial default).^1^^1^NB: Many articles refer to initial loss to follow-up as ‘initial default’. Per MacPherson et al. [74] and Zachariah et al. (2012) the authors have chosen to use the terminology ‘initial loss to follow-up’ as ‘default’ is often considered pejorative and a victimizing word for individuals with TB. Fourteen articles described geography and distance as barriers to TB care [17,22,27,28,31,37–39,49,51,55,57,60,63]. Facilities in rural areas with improved TB diagnostic and treatment capacity could reduce diagnostic and treatment delays [57]. Ten articles described laboratory and clinical services offered, such as the impact of drug-resistance testing, and point-of-care testing as barriers to treatment initiation [18,27,31,44,48,49,55,57,60,63]. Healthcare structures, such as the level of service at which TB disease is diagnosed, where treatment can be initiated, and where treatment can be sustained, were also described as a barrier to treatment initiation in 17 articles [22,26,28,29,31,32,37,43,44,46,47,49,50,57,60–62,63]. For example, Sendigire (2010) found that over 90% of patients visited more than one healthcare provider and had an average of four visits prior to receiving a diagnosis of TB and less than 5% of patients were diagnosed on their first visit to a healthcare provider [39]. Furthermore, fixed primary healthcare centers (PHCs) and mobile clinics were evaluated to assess the collection and recording process of results, with 86% of patients at PHCs having two sputum samples recorded while only 69% of mobile clinics reported two sputum results [32]. All but two articles described a health services delay or the quality of care as a barrier to treatment initiation which in some instances prevented treatment initiation [37,62]. Overall, health service delays often resulted from centralization of healthcare as well as fragmentation between referrals, diagnostic delays, and hierarchical structures of healthcare [43,60,63].

Additionally, initial loss to follow-up was considered a structural barrier. When a patient is diagnosed but never initiates TB treatment, often it is the health system’s failure to report diagnostic results or make timely follow-up, rather than a patient’s unwillingness to start therapy, that is responsible. However, a mixture of lost laboratory results, long wait times between sputum collection and final culture results, and the number of providers and technicians handling the specimen can all contribute to this initial loss to follow-up [33]. Seven studies specifically measured patients with initial loss to follow-up, with rates as high as 40% [32–34,47,49,53,63]. No article described loss to follow-up as a function of patient age; however, Yassin et al. (2013) disaggregated symptomatic patients and smear positive pulmonary TB patients by age and sex for their community-based TB intervention, which benefitted women, children, and vulnerable groups the most [49].

The three studies of child-only cohorts addressed delays for children and the complexity and value of culture confirmation for children with TB. Waiting for culture confirmation prior to treatment can greatly increase delays in children compared to initiating treatment when a clinical diagnosis is made (median 1 day with clinical diagnosis versus median 40 days with culture diagnosis) [18]. Additionally, treatment is delayed in children when an adult source is unknown (median 58 days when adult source is known versus 123 days without a source) [19]. Follow-up in urban squatter communities was difficult, causing children in these locations to have significantly worse tracing than children in urban settled areas, rural agriculture areas, and rural settled areas [17]. All of these child-only cohort studies were conducted in South Africa.

### Health-seeking behavior as a barrier to TB treatment initiation

Health-seeking behavior is complex and is influenced by knowledge, attitudes, beliefs, and accessibility of care pathways. Nineteen articles examined health-seeking behaviors specific to barriers and the subsequent delays they may cause (Supplement 4) [20,21,23–25,28–30,35,36,38,39,42,43,51,52,55,59,61]. These behaviors include seeking care from formal and informal sectors, private and public healthcare providers, traditional healers, pharmacies or drug retailers, and private clinics. All 19 articles describing health-seeking behaviors considered patient-level behaviors (i.e. patient knowledge, attitudes, behaviors, and decisions regarding care pathways). Only three studies also considered system-level factors, including stigmatization from healthcare providers as well as provider knowledge of TB acting as barriers against patients seeking care [25,28,36].

All but one article in this category addressed knowledge, attitudes, or beliefs about TB [30]. All articles but four discussed patient care pathways [20,36,51,61]. Eight studies were qualitative [20,21,23–25,28,36,42], 10 were quantitative [29,30,35,38,39,43,51,52,55,59], and 1 used mixed methods [61]. Patient knowledge of the causes of TB varied between 0% and 63% of patients having ‘good’ knowledge of TB [25,28]. Stigma was often cited in regards to patients’ attitudes and beliefs about TB, and patients often associated TB with HIV/AIDS [28,61]. Some studies identified patients who lacked knowledge of TB, yet delayed seeking care because of fear of potential disease or its attached stigma [21,42]. Preference for traditional medicine was also cited [30,42,59]. In addition, parents voiced concern about their children being exposed to increased infections while waiting to be seen at healthcare facilities, which significantly contributed to children not receiving care (OR [odds ratio] 2.45, 95% CI [confidence interval] 1.07–5.60, *p* = 0.03) [20].

Patients’ decisions of where to first seek care were influenced by numerous factors. These decisions were made by patients themselves, by close family members, or by healthcare workers [21,24]. Distance from home and mode of transport also was a factor in where to seek care, with closer proximity to home and walking distance preferred [21]. However, patients who visited health centers, private facilities, and health posts were more likely to experience delays compared to those who visited hospitals [57]. Eastwood and Hill (2004) reported that patients who consulted with pharmacies had diagnostic delays of around one month, while patients who consulted traditional healers had delays of several months. Many studies cited multiple care provider visits prior to diagnosis and appropriate treatment of TB with one study citing upwards of six facilities being visited [28]. In cases where the mother was the source of multi-drug resistant tuberculosis (MDR-TB) infection, children were almost four times less likely to receive MDR-TB care than if the mother was not the source of infection (OR 3.78, 95% CI 1.29–11.1, *p *= 0.02) [20]. Thus, children with close household contact to MDR-TB received delayed care.

## Discussion

This review suggests that more research in younger populations is urgently needed related to barriers to TB treatment. Previous studies have described delayed treatment for children and youth with HIV or those with TB [8,64,65]. However, there is a paucity of research specifically pertaining to TB treatment initiation in youth with or without HIV [6,66]. More specifically, the epidemiology of drug-resistant TB (DR-TB) and DR-TB/HIV coinfection and treatment in youth remains unclear. Children and youth are now priority populations in TB research [67]. Thus, there should be increased research specific to children and youth. Only four of the reviewed studies were specific to children and youth. These four studies focused on barriers to timely treatment initiation with a pediatric lens. Interestingly, most of the same barriers existed for adults and children with the exception of increased social, logistic, and cultural factors contributing to pediatric non-attendance at clinics including the mother also being ill, which likely would not affect adults as much as children [20].

### Cost barriers

Both direct and indirect costs pose barriers to TB treatment initiation for individuals of all ages in SSA. Additionally, intangible costs and costs associated with caretaker burden influence when and where someone seeks TB treatment. Catastrophic medical expenses and poverty can delay, and potentially prevent, individuals from initiating TB treatment [4]. Therefore, community-based TB treatment initiation, and grants and other resources for transportation, nutrition, and financial services such as through National TB Programs, may enable earlier and more adequate TB treatment. Interestingly, cost barriers were not found to be reported until 2010, suggesting that economic studies have only recently become important to researchers in SSA.

Costs unique to child and youth TB treatment initiation must be studied more specifically. For example, pediatric drug formulations are often more expensive than standard adult formulations, which may create additional financial barriers for either families if paying out of pocket or health systems if provided by National TB Programs [68]. Additionally, intangible costs such as lost days of school for a child and missed days of work for a parent double the burden. No studies assessed caretaker costs among pediatric cohorts; two studies evaluating these intangible costs only included individuals 15 years and older [40,41]. In addition, pediatric TB specialists are often in more centralized or urban areas, creating a heavier financial burden for younger children who must travel further from home, and often must be accompanied by an adult.

### Infrastructure barriers

Delays were observed in children from rural or farming areas, and when errors were made by treating physicians [17]. Additionally, lack of point-of-care laboratory capacity and the lack of ability to perform certain diagnostic testing (such as cultures and drug susceptibility testing) often caused treatment delays, especially for drug-resistant TB [18]. Thus, starting same-day empiric treatment prior to culture results can greatly reduce barriers to initiating treatment.

Improving health system infrastructure through integrating TB services into existing programs was found to be critical for all ages. Deficiencies of health systems were apparent across multiple settings; application of recommended TB/HIV integration programs should be utilized [69]. The level of care at which TB services were available influenced where patients first sought treatment, with centralized services often delaying or inhibiting treatment initiation [37,43].

Geographic barriers are common and limit access to treatment. Thus, developing diagnostic and treatment options in rural areas, and training healthcare providers on the signs and symptoms of TB, could remove some geographic barriers to TB treatment initiation [39,63]. Community-based interventions have been shown to be cost-effective, such as leveraging health extension workers to educate communities on sanitation and hygiene, debunking TB and HIV myths and lessening stigma, as well as in conducting screenings [34,37]. While the public sector is by no means a panacea for TB treatment initiation, it could actively engage and educate traditional practitioners, the private sector, and community partners in order to improve services to patients closer to their homes and at more affordable costs [70,71]. Overall, practical, effective policies to strengthen health systems can create enormous benefits in TB care for all ages, and children in particular.

### Health-seeking behaviors

Educating both providers and patients about TB and the importance of timely, effective treatment can greatly improve outcomes. Although Edginton et al. (2005) found that knowledge of TB was good in 63% of patients, 51% of patients were unaware of the cause of TB. Thus, ‘good’ knowledge should be interpreted with caution, especially when assessing knowledge across studies using different measures. Similarly, two studies found TB stigma was associated with stigma against HIV in South Africa and Zambia, two high HIV prevalence countries [28,61]. Thus, country context also affected health-seeking behaviors. Specific to children, when the source of M.DR-TB was from the mother, clinic follow-up attendance was worse [20]. Providing patient-centered TB care is one way in which some barriers can be removed from accessing treatment initiation and improving clinic follow-up after diagnosis [20,72].

Chaotic and uncoordinated services can cause delays and increase costs for patients with TB. A coordinated National TB Program can streamline services, thus improving general health education, promoting TB prevention, and regulating healthcare providers in both the private and public sectors. On the other hand, decentralized care provided through community-based organizations may facilitate easier, more cost-effective care pathways [37]. Decreasing the number of providers that patients visit prior to an accurate diagnosis and effective treatment is one way in which to facilitate more timely treatment initiation. These strategies would have even greater impact upon child and youth cases, though more research in this area is needed.

### Limitations

Due to the inclusion of all study designs, there was great heterogeneity of these studies, therefore neither a pooled analysis nor a meta-analysis was conducted and no summary measures (e.g. effect size) for specific interventions could be determined. Further, inconsistencies in cost measures were found to be a limitation, as well as differences across countries. Not all studies captured data in the same manner, nor used the same definitions or timeframes for analysis. However, most articles noted in their limitations the difficulty of getting accurate income data and verifying direct and indirect costs in low-income settings. Although we undertook a systematic literature search, some studies meeting our inclusion criteria may have been missed.

## Conclusion

Many patient- and system-level barriers to TB treatment initiation exist among children and youth in SSA; however, through systematic review of the literature, these barriers are more fully described for adults. To our knowledge, no study has correlated barriers to treatment initiation with patient outcomes, and more evidence in this area could benefit TB prevention – and could thereby save lives [73]. The specific needs of children and youth should be prioritized in research, particularly around enhanced infrastructure such as early diagnosis and treatment initiation and community- and patient-centered approaches. We recommend more standardized language to describe barriers to TB treatment initiation within the TB research and advocacy community, in order to allow for more unified, collective, and powerful action (Table 1) [72]. Addressing both patient- and system-level barriers is vital to improving patient outcomes, especially among young populations.

## Supplementary Material

Supplemental Data

## Paper context

Effective, age-specific treatment for tuberculosis in sub-Saharan Africa is vital to disease management and prevention of transmission in communities. Barriers to tuberculosis treatment initiation are well-documented in mixed age groups; however, this systematic review reveals that insufficient literature assesses barriers specific to children and youth. Therefore, this paper adds valuable insights into specific barriers to tuberculosis treatment initiation in sub-Saharan Africa, emphasizing the need for additional research in vulnerable pediatric populations.
