# Supplementary material for: Barriers to initiating tuberculosis treatment in sub-Saharan Africa: a systematic review focused on children and youth
Source: Glob Health Action. 2017 Jun 9;10(1):1290317. doi: 10.1080/16549716.2017.1290317 (PMC5496082; doi:10.1080/16549716.2017.1290317)
Supplement: Supplemental Data [file zgha_a_1290317_sm6643.docx]

Supplement 1. Protocol

**Research Question:** What are the patient- and system-level barriers to treatment initiation for pediatric and youth tuberculosis care in sub-Saharan Africa?

| Exclusion Criteria | Explanation: |
| --- | --- |
| 1. Focus Not TB | Exclude if it’s about a disease and TB is only a sub-population (i.e. mental health and TB, HIV and those co-infected with TB) |
| 2. Not SSA | Exclude Europe, MENA, Asia, Americas, Africans outside Africa |
| 3. Not pediatrics | Must include 0-24 years, exclude if only age 18+ |
| 4. Not barriers | Must include a specific barrier (i.e. delay, treatment initiation, access to care, location of services, cost, adverse drugs, stigma, fear, perceptions of treatment, etc) |
| 5. Not original research | Exclude reviews, case studies |
| 6. Not English | Exclude if article is not available for full review in English |

Supplement 2. Full Database Search

Databases: Pubmed, Embase, CINAHL, African Medicus Index, Global health, SCOPUS

PubMed

| Set # |  | Results |
| --- | --- | --- |
| 1 | "Tuberculosis"[Mesh] OR "tuberculosis"[ti] OR "TB"[ti] | 189261 |
| 2 | "Africa South of the Sahara"[Mesh] OR Africa[tiab] OR African[tiab] OR angola[tiab] OR benin[tiab] OR botswana[tiab] OR burkina faso[tiab] OR burundi[tiab] OR cameroon[tiab] OR cape verde[tiab] OR central african republic[tiab] OR chad[tiab] OR comoros[tiab] OR congo[tiab] OR cote d'ivoire[tiab] OR ivory coast[tiab] OR congo[tiab] OR zaire[tiab] OR Djibouti[tiab] OR equatorial guinea[tiab] OR ethiopia[tiab] OR eritrea[tiab] OR gabon[tiab] OR gambia[tiab] OR ghana[tiab] OR guinea[tiab] OR guinea-bissau[tiab] OR kenya[tiab] OR lesotho[tiab] OR liberia[tiab] OR madagascar[tiab] OR malawi[tiab] OR mali[tiab] OR mauritania[tiab] OR mauritius[tiab] OR Mayotte[tiab] OR mozambique[tiab] OR namibia[tiab] OR niger[tiab] OR nigeria[tiab] OR reunion[tiab] OR rwanda[tiab] OR sahara[tiab] OR saint Helena[tiab] OR sao tome[tiab] OR senegal[tiab] OR seychelles[tiab] OR sierra leone[tiab] OR somalia[tiab] OR south africa[tiab] OR sudan[tiab] OR swaziland[tiab] OR togo[tiab] OR tanzania[tiab] OR uganda[tiab] OR zambia[tiab] OR zimbabwe[tiab] | 402257 |
| 3 | "Delivery of Health Care/supply and distribution"[Mesh] OR "Pharmaceutical Preparations/supply and distribution"[Mesh] OR "Pharmaceutical Preparations/economics"[Mesh] OR "Antitubercular Agents/adverse effects"[Mesh] OR "Antitubercular Agents/economics"[Mesh] OR "Antitubercular Agents/supply and distribution"[Mesh] OR "Attitude to Health"[Mesh] OR "Patient Dropouts"[Mesh] OR "Motivation"[Mesh] OR "Time-to-Treatment"[Mesh] OR "Time Factors"[Mesh] OR "Social Stigma"[Mesh] OR "Social Support"[Mesh] OR dropout[tiab] OR dropouts[tiab] OR refuse[tiab] OR refusal[tiab] OR refused[tiab] OR initiate[tiab] OR initiated[tiab] OR initiation[tiab] OR non-initiation[tiab] OR noninitiation[tiab] OR facilitate[tiab] OR facilitator[tiab] OR facilitated[tiab] OR facilitators[tiab] OR barrier[tiab] OR barriers[tiab] OR uptake[tiab] OR motivat*[tiab] OR delay[tiab] OR delayed[tiab] OR delays[tiab] OR time[tiab] OR supply[tiab] OR stigma[tiab] OR support[tiab] OR economics[tiab] OR pathway[tiab] OR pathways[tiab] OR "side effect"[tiab] OR "side effects"[tiab] OR "adverse effect"[tiab] OR "adverse effects"[tiab] | 5558395 |
| 4 | #1 AND #2 AND #3 | **2937** |
| 5 | "Focus Groups"[Mesh] OR "Questionnaires"[Mesh] OR "Qualitative research"[Mesh] OR "Observational Study" [Publication Type] OR "Interview" [Publication Type] OR "personal narratives as topic"[Mesh] OR "interviews as topic"[Mesh] OR "Narration"[Mesh] OR "Nursing Research"[Mesh] OR "Anecdotes as Topic"[Mesh] OR "Tape Recording"[Mesh] OR "Observational Study as Topic"[Mesh] OR "semi-structured"[tiab] OR semistructured[tiab] OR unstructured[tiab] OR informal[tiab] OR "in-depth"[tiab] OR indepth[tiab] OR "face-to-face"[tiab] OR structured[tiab] OR interview*[tiab] OR discussion[tiab] OR discussions[tiab] OR questionnaire[tiab] OR questionnaires[tiab] OR survey[tiab] OR surveys[tiab] OR surveyed[tiab] OR "focus group"[tiab] OR "focus groups"[tiab] OR qualitative[tiab] OR ethnography[tiab] OR ethnographic[tiab] OR fieldwork[tiab] OR "field work"[tiab] OR "key informant"[tiab] OR randomized controlled trial[pt] OR controlled clinical trial[pt] OR randomized[tiab] OR randomised[tiab] OR randomization[tiab] OR randomisation[tiab] OR placebo[tiab] OR drug therapy[sh] OR randomly[tiab] OR trial[tiab] OR groups[tiab] OR Clinical trial[pt] OR “clinical trial”[tiab] OR “clinical trials”[tiab] OR "evaluation studies"[Publication Type] OR "evaluation studies as topic"[MeSH Terms] OR "evaluation study"[tiab] OR evaluation studies[tiab] OR "intervention studies"[MeSH Terms] OR "intervention study"[tiab] OR "intervention studies"[tiab] OR "case-control studies"[MeSH Terms] OR "case-control"[tiab] OR "cohort studies"[MeSH Terms] OR cohort[tiab] OR "longitudinal studies"[MeSH Terms] OR "longitudinal”[tiab] OR longitudinally[tiab] OR "prospective"[tiab] OR prospectively[tiab] OR "retrospective studies"[MeSH Terms] OR "retrospective"[tiab] OR "follow up"[tiab] OR "comparative study"[Publication Type] OR "comparative study"[tiab] | 7770042 |
| 6 | #4 AND #5 | 2258 |
| 7 | "Pediatrics"[Mesh] OR "Adolescent"[Mesh] OR "Child"[Mesh] OR "Infant"[Mesh] OR "Young Adult"[Mesh] OR child[tiab] OR children[tiab] OR infant[tiab] OR infants[tiab] OR pediatric[tiab] OR teenager[tiab] OR teenagers[tiab] OR teenaged[tiab] OR teen[tiab] OR teens[tiab] OR "young adult"[tiab] OR "young adults"[tiab] OR adolescent[tiab] OR adolescents[tiab] OR youth[tiab] | 3496121 |
| 8 | #6 AND #7 [as of 1:45pm on 10.26.15] | 923 |
| 9 | NOT (animals[mh] NOT humans[mh]) NOT (Editorial[pt] OR Letter[pt] OR Case Reports[pt] OR Comment[pt]) | **899** |

Embase:

| Set # |  | Results |
| --- | --- | --- |
| 1 | 'tuberculosis'/exp OR tuberculosis:ti OR TB:ti | 266395 |
| 2 | 'Africa south of the Sahara'/exp OR Africa:ti,ab OR African:ti,ab OR angola:ti,ab OR benin:ti,ab OR botswana:ti,ab OR 'burkina faso':ti,ab OR burundi:ti,ab OR cameroon:ti,ab OR 'cape verde':ti,ab OR 'central african republic':ti,ab OR chad:ti,ab OR comoros:ti,ab OR congo:ti,ab OR 'cote divoire':ti,ab OR 'ivory coast':ti,ab OR congo:ti,ab OR zaire:ti,ab OR Djibouti:ti,ab OR 'equatorial guinea':ti,ab OR ethiopia:ti,ab OR eritrea:ti,ab OR gabon:ti,ab OR gambia:ti,ab OR ghana:ti,ab OR guinea:ti,ab OR 'guinea bissau':ti,ab OR kenya:ti,ab OR lesotho:ti,ab OR liberia:ti,ab OR madagascar:ti,ab OR malawi:ti,ab OR mali:ti,ab OR mauritania:ti,ab OR mauritius:ti,ab OR Mayotte:ti,ab OR mozambique:ti,ab OR namibia:ti,ab OR niger:ti,ab OR nigeria:ti,ab OR reunion:ti,ab OR rwanda:ti,ab OR sahara:ti,ab OR 'saint Helena':ti,ab OR 'sao tome':ti,ab OR senegal:ti,ab OR seychelles:ti,ab OR 'sierra leone':ti,ab OR somalia:ti,ab OR 'south africa':ti,ab OR sudan:ti,ab OR swaziland:ti,ab OR togo:ti,ab OR tanzania:ti,ab OR uganda:ti,ab OR zambia:ti,ab OR zimbabwe:ti,ab | 491851 |
| 3 | 'drug'/exp/dd_pe OR 'drug industry'/exp OR 'health care distribution'/exp OR 'tuberculostatic agent'/exp/dd_pe,dd_ae OR 'attitude to health'/exp OR 'patient attitude'/exp OR 'patient dropouts'/exp OR 'motivation'/exp OR 'time to treatment'/exp OR 'time'/exp OR 'stigma'/exp OR 'social stigma'/exp OR 'social support'/exp OR dropout:ti,ab OR dropouts:ti,ab OR refuse:ti,ab OR refusal:ti,ab OR refused:ti,ab OR initiate:ti,ab OR initiated:ti,ab OR initiation:ti,ab OR 'non initiation':ti,ab OR noninitiation:ti,ab OR facilitate:ti,ab OR facilitator:ti,ab OR facilitated:ti,ab OR facilitators:ti,ab OR barrier:ti,ab OR barriers:ti,ab OR uptake:ti,ab OR delay:ti,ab OR delayed:ti,ab OR delays:ti,ab OR time:ti,ab OR supply:ti,ab OR stigma:ti,ab OR support:ti,ab OR economics:ti,ab OR pathway:ti,ab OR pathways:ti,ab OR 'side effect':ti,ab OR 'side effects':ti,ab OR 'adverse effect':ti,ab OR 'adverse effects':ti,ab OR motivat*:ab,ti | 6815184 |
| 4 | #1 AND #2 AND #3 | **4516** |
| 5 | 'information processing'/exp OR 'questionnaire'/exp OR 'qualitative research'/exp OR 'observational study'/exp OR 'interview'/exp OR 'literature'/exp OR 'verbal communication'/exp OR 'nursing research'/exp OR 'recording'/exp OR 'semi-structured':ti,ab OR semistructured:ti,ab OR unstructured:ti,ab OR informal:ti,ab OR 'in-depth':ti,ab OR indepth:ti,ab OR 'face-to-face':ti,ab OR structured:ti,ab OR interview*:ti,ab OR discussion:ti,ab OR discussions:ti,ab OR questionnaire:ti,ab OR questionnaires:ti,ab OR survey:ti,ab OR surveys:ti,ab OR surveyed:ti,ab OR 'focus group':ti,ab OR 'focus groups':ti,ab OR qualitative:ti,ab OR ethnography:ti,ab OR ethnographic:ti,ab OR fieldwork:ti,ab OR 'field work':ti,ab OR 'key informant':ti,ab OR 'randomized controlled trial'/exp OR 'crossover procedure'/exp OR 'double blind procedure'/exp OR 'single blind procedure'/exp OR random*:ab,ti OR factorial*:ab,ti OR crossover*:ab,ti OR (cross NEAR/1 over*):ab,ti OR placebo*:ab,ti OR (doubl* NEAR/1 blind*):ab,ti OR (singl* NEAR/1 blind*):ab,ti OR assign*:ab,ti OR allocat*:ab,ti OR volunteer*:ab,ti OR 'clinical study'/exp OR ‘clinical trial’:ti,ab OR ‘clinical trials’:ti,ab OR 'controlled study'/exp OR 'evaluation'/exp OR ‘evaluation study’:ab,ti OR ‘evaluation studies’:ab,ti OR ‘intervention study’:ab,ti OR ‘intervention studies’:ab,ti OR ‘case control’:ab,ti OR 'cohort analysis'/exp OR cohort:ab,ti OR longitudinal*:ab,ti OR prospective:ab,ti OR prospectively:ab,ti OR retrospective:ab,ti OR 'follow up'/exp OR ‘follow up’:ab,ti OR 'comparative study'/exp OR ‘comparative study’:ab,ti OR ‘comparative studies’:ab,ti | 13904707 |
| 6 | #4 AND #5 | 3293 |
| 7 | 'pediatrics'/exp OR 'adolescent'/exp OR 'child'/exp OR 'infant'/exp OR 'young adult'/exp OR child:ti,ab OR children:ti,ab OR infant:ti,ab OR infants:ti,ab OR pediatric:ti,ab OR teenager:ti,ab OR teenagers:ti,ab OR teenaged:ti,ab OR teen:ti,ab OR teens:ti,ab OR 'young adult':ti,ab OR 'young adults':ti,ab OR adolescent:ti,ab OR adolescents:ti,ab OR youth:ti,ab | 3546508 |
| 8 | #6 AND #7 | 960 |
| 9 | #8 AND [embase]/lim NOT [medline]/lim | 253 |
|  | NOT (‘case report’/exp OR ‘case study’/exp OR ‘editorial’/exp OR ‘letter’/exp OR ‘note’/exp) AND (‘humans’/lim) | **217**  **as of 10/26/15** |

CINAHL:

| Set # |  | Results |
| --- | --- | --- |
| 1 | (MH "Tuberculosis+") OR TI (Tuberculosis OR TB) | 17151 |
| 2 | (MH "Africa South of the Sahara+") OR TI (Africa OR African OR angola OR benin OR botswana OR burkina faso OR burundi OR cameroon OR cape verde OR central african republic OR chad OR comoros OR congo OR cote d'ivoire OR ivory coast OR congo OR zaire OR Djibouti OR equatorial guinea OR ethiopia OR eritrea OR gabon OR gambia OR ghana OR guinea OR guinea-bissau OR kenya OR lesotho OR liberia OR madagascar OR malawi OR mali OR mauritania OR mauritius OR Mayotte OR mozambique OR namibia OR niger OR nigeria OR reunion OR rwanda OR sahara OR saint Helena OR sao tome OR senegal OR seychelles OR sierra leone OR somalia OR south africa OR sudan OR swaziland OR togo OR tanzania OR uganda OR zambia OR zimbabwe) OR AB (Africa OR African OR angola OR benin OR botswana OR burkina faso OR burundi OR cameroon OR cape verde OR central african republic OR chad OR comoros OR congo OR cote d'ivoire OR ivory coast OR congo OR zaire OR Djibouti OR equatorial guinea OR ethiopia OR eritrea OR gabon OR gambia OR ghana OR guinea OR guinea-bissau OR kenya OR lesotho OR liberia OR madagascar OR malawi OR mali OR mauritania OR mauritius OR Mayotte OR mozambique OR namibia OR niger OR nigeria OR reunion OR rwanda OR sahara OR saint Helena OR sao tome OR senegal OR seychelles OR sierra leone OR somalia OR south africa OR sudan OR swaziland OR togo OR tanzania OR uganda OR zambia OR zimbabwe) | 72414 |
| 3 | (MH "Health Care Delivery+/UT") OR (MH "Drugs+/SD/EC") OR (MH "Antitubercular Agents+/AE/EC/SD") OR (MH "Attitude to Health+") OR (MH "Patient Dropouts") OR (MH "Motivation+") OR (MH "Time Factors") OR (MH "Treatment Delay") OR (MH "Stigma") OR (MH "Support, Psychosocial+") OR TI (dropout OR dropouts OR refuse OR refusal OR refused OR initiate OR initiated OR initiation OR non-initiation OR noninitiation OR facilitate OR facilitator OR facilitated OR facilitators OR barrier OR barriers OR uptake OR delay OR delayed OR delays OR time OR supply OR stigma OR support OR economics OR pathway OR pathways OR "side effect" OR "side effects" OR "adverse effect" OR "adverse effects" OR motivat*) OR AB (dropout OR dropouts OR refuse OR refusal OR refused OR initiate OR initiated OR initiation OR non-initiation OR noninitiation OR facilitate OR facilitator OR facilitated OR facilitators OR barrier OR barriers OR uptake OR delay OR delayed OR delays OR time OR supply OR stigma OR support OR economics OR pathway OR pathways OR "side effect" OR "side effects" OR "adverse effect" OR "adverse effects" OR motivat*) | 833820 |
| 4 | #1 AND #2 AND #3 | **469** |
| 5 | (MH "Focus Groups") OR (MH "Questionnaires+") OR (MH "Qualitative Studies+") OR (MH "Nonexperimental Studies+") OR PT interview OR (MH "Storytelling+") OR (MH "Interviews+") OR (MH "Narratives") OR (MH "Research, Nursing") OR (MH "Nursing Administration Research") OR (MH "Clinical Nursing Research") OR (MH "Nursing Practice, Research-Based") OR (MH "Videorecording+") OR (MH "Audiorecording") OR TI ("semi-structured" OR semistructured OR unstructured OR informal OR "in-depth" OR indepth OR "face-to-face" OR structured OR interview* OR discussion OR discussions OR questionnaire OR questionnaires OR survey OR surveys OR surveyed OR "focus group" OR "focus groups" OR qualitative OR ethnography OR ethnographic OR fieldwork OR "field work" OR "key informant" OR motivat*) OR AB ("semi-structured" OR semistructured OR unstructured OR informal OR "in-depth" OR indepth OR "face-to-face" OR structured OR interview* OR discussion OR discussions OR questionnaire OR questionnaires OR survey OR surveys OR surveyed OR "focus group" OR "focus groups" OR qualitative OR ethnography OR ethnographic OR fieldwork OR "field work" OR "key informant" OR motivat*) OR PT randomized controlled trial OR PT clinical trial OR TI (randomized OR randomised OR randomization OR randomisation OR placebo OR randomly OR trial OR groups OR “clinical trial” OR “clinical trials”) OR AB (randomized OR randomised OR randomization OR randomisation OR placebo OR randomly OR trial OR groups OR “clinical trial” OR “clinical trials”) OR (MH "Evaluation Research+") OR TI ("evaluation study" OR "evaluation studies") OR AB ("evaluation study" OR "evaluation studies") OR (MH "Experimental Studies+") OR TI ("intervention study" OR "intervention studies" OR "case-control" OR cohort OR "longitudinal” OR longitudinally OR "prospective" OR prospectively) OR AB ("intervention study" OR "intervention studies" OR "case-control" OR cohort OR "longitudinal” OR longitudinally OR "prospective" OR prospectively) OR (MH "Retrospective Design") OR TI ("retrospective" OR "follow up") OR AB ("retrospective" OR "follow up") OR (MH "Comparative Studies") OR TI ("comparative study") OR AB ("comparative study") | 1651496 |
| 6 | #4 AND #5 | 376 |
| 7 | (MH "Pediatrics+") OR (MH "Adolescence+") OR (MH "Child+") OR (MH "Infant+") OR (MH "Young Adult") OR TI (child OR children OR infant OR infants OR pediatric OR teenager OR teenagers OR teenaged OR teen OR teens OR "young adult" OR "young adults" OR adolescent OR adolescents OR youth) OR AB (child OR children OR infant OR infants OR pediatric OR teenager OR teenagers OR teenaged OR teen OR teens OR "young adult" OR "young adults" OR adolescent OR adolescents OR youth) | 804054 |
| 8 | #6 AND #7 | **113**  **as of 10.26.15** |

Global Health:

| Set # |  | Results |
| --- | --- | --- |
| 1 | DE "tuberculosis" OR DE "extrapulmonary tuberculosis" OR DE "miliary tuberculosis" OR TI ("tuberculosis" OR "TB") | 36773 |
| 2 | DE "Africa South of Sahara" OR DE "Central Africa" OR DE "East Africa" OR DE "Sahel" OR DE "Southern Africa" OR DE "West Africa" OR TI (Africa OR African OR angola OR benin OR botswana OR burkina faso OR burundi OR cameroon OR cape verde OR central african republic OR chad OR comoros OR congo OR cote d'ivoire OR ivory coast OR congo OR zaire OR Djibouti OR equatorial guinea OR ethiopia OR eritrea OR gabon OR gambia OR ghana OR guinea OR guinea-bissau OR kenya OR lesotho OR liberia OR madagascar OR malawi OR mali OR mauritania OR mauritius OR Mayotte OR mozambique OR namibia OR niger OR nigeria OR reunion OR rwanda OR sahara OR saint Helena OR sao tome OR senegal OR seychelles OR sierra leone OR somalia OR south africa OR sudan OR swaziland OR togo OR tanzania OR uganda OR zambia OR zimbabwe) OR AB (Africa OR African OR angola OR benin OR botswana OR burkina faso OR burundi OR cameroon OR cape verde OR central african republic OR chad OR comoros OR congo OR cote d'ivoire OR ivory coast OR congo OR zaire OR Djibouti OR equatorial guinea OR ethiopia OR eritrea OR gabon OR gambia OR ghana OR guinea OR guinea-bissau OR kenya OR lesotho OR liberia OR madagascar OR malawi OR mali OR mauritania OR mauritius OR Mayotte OR mozambique OR namibia OR niger OR nigeria OR reunion OR rwanda OR sahara OR saint Helena OR sao tome OR senegal OR seychelles OR sierra leone OR somalia OR south africa OR sudan OR swaziland OR togo OR tanzania OR uganda OR zambia OR zimbabwe) | 176608 |
| 3 | DE "antituberculous agents" OR DE "ethambutol" OR DE "ethionamide" OR DE "minocycline" OR DE "pyrazinamide" OR DE "thioacetazone" OR DE "attitudes" OR DE "consumer attitudes" OR DE "motivation" OR DE "timing" OR DE "time lag" OR DE "social stigma" OR DE "support systems" OR DE "personal support networks" OR TI (dropout OR dropouts OR refuse OR refusal OR refused OR initiate OR initiated OR initiation OR non-initiation OR noninitiation OR facilitate OR facilitator OR facilitated OR facilitators OR barrier OR barriers OR uptake OR delay OR delayed OR delays OR time OR supply OR stigma OR support OR economics OR pathway OR pathways OR "side effect" OR "side effects" OR "adverse effect" OR "adverse effects" OR supply OR motivat*) OR AB (dropout OR dropouts OR refuse OR refusal OR refused OR initiate OR initiated OR initiation OR non-initiation OR noninitiation OR facilitate OR facilitator OR facilitated OR facilitators OR barrier OR barriers OR uptake OR delay OR delayed OR delays OR time OR supply OR stigma OR support OR economics OR pathway OR pathways OR "side effect" OR "side effects" OR "adverse effect" OR "adverse effects" OR supply OR motivat*) | 694687 |
| 4 | #1 AND #2 AND #3 | **1811** |
| 5 | DE "questionnaires" OR DE "qualitative analysis" OR DE "qualitative techniques" OR DE "interviews" OR DE "medical research" OR DE "video recordings" OR DE "videotapes" OR TI ("semi-structured" OR semistructured OR unstructured OR informal OR "in-depth" OR indepth OR "face-to-face" OR structured OR interview* OR discussion OR discussions OR questionnaire OR questionnaires OR survey OR surveys OR surveyed OR "focus group" OR "focus groups" OR narration OR narrative OR narratives OR anecdote OR anecdotes OR observation OR observations OR observational OR qualitative OR ethnography OR ethnographic OR fieldwork OR "field work" OR "key informant") OR AB ("semi-structured" OR semistructured OR unstructured OR informal OR "in-depth" OR indepth OR "face-to-face" OR structured OR interview* OR discussion OR discussions OR questionnaire OR questionnaires OR survey OR surveys OR surveyed OR "focus group" OR "focus groups" OR narration OR narrative OR narratives OR anecdote OR anecdotes OR observation OR observations OR observational OR qualitative OR ethnography OR ethnographic OR fieldwork OR "field work" OR "key informant") OR DE "clinical trials" OR DE "randomized controlled trials" OR TI (randomized OR randomised OR randomization OR randomisation) OR AB (randomized OR randomised OR randomization OR randomisation) OR TI (randomly OR trial OR groups OR “clinical trial” OR “clinical trials”) OR AB (randomly OR trial OR groups OR “clinical trial” OR “clinical trials”) OR DE "evaluation" OR TI ("evaluation study" OR evaluation studies) OR AB ("evaluation study" OR evaluation studies) OR DE "intervention" OR TI ("intervention study" OR "intervention studies" OR "case-control" OR cohort studies OR cohort OR "longitudinal” OR longitudinally OR "prospective" OR prospectively OR retrospective OR "follow up" OR "comparative study") OR AB ("intervention study" OR "intervention studies" OR "case-control" OR cohort studies OR cohort OR "longitudinal” OR longitudinally OR "prospective" OR prospectively OR retrospective OR "follow up" OR "comparative study") OR DE "longitudinal studies" | 698051 |
| 6 | #4 AND #5 | 1811 |
| 7 | DE "paediatrics" OR DE "adolescents" OR DE "children" OR DE "preschool children" OR DE "school children" OR DE "infants" OR DE "young adults" OR TI (child OR children OR infant OR infants OR pediatric OR teenager OR teenagers OR teenaged OR teen OR teens OR "young adult" OR "young adults" OR adolescent OR adolescents OR youth) OR AB (child OR children OR infant OR infants OR pediatric OR teenager OR teenagers OR teenaged OR teen OR teens OR "young adult" OR "young adults" OR adolescent OR adolescents OR youth) | 331165 |
| 8 | #6 AND #7 | **292**  as of 10.26.15 |

SCOPUS:

| Set # |  | Results |
| --- | --- | --- |
| 1 | TITLE (tuberculosis OR tb) | 139433 |
| 2 | TITLE-ABS-KEY (Africa OR African OR angola OR benin OR botswana OR "burkina faso" OR burundi OR cameroon OR "cape verde" OR "central african republic" OR chad OR comoros OR congo OR "cote d'ivoire" OR "ivory coast" OR congo OR zaire OR Djibouti OR "equatorial guinea" OR ethiopia OR eritrea OR gabon OR gambia OR ghana OR guinea OR "guinea Bissau" OR kenya OR lesotho OR liberia OR madagascar OR malawi OR mali OR mauritania OR mauritius OR Mayotte OR mozambique OR namibia OR niger OR nigeria OR reunion OR rwanda OR sahara OR "saint Helena" OR "sao tome" OR senegal OR seychelles OR "sierra leone" OR somalia OR "south Africa" OR sudan OR swaziland OR togo OR tanzania OR uganda OR zambia OR zimbabwe) | 910333 |
| 3 | TITLE-ABS-KEY (dropout OR dropouts OR refuse OR refusal OR refused OR initiate OR initiated OR initiation OR non-initiation OR noninitiation OR facilitate OR facilitator OR facilitated OR facilitators OR barrier OR barriers OR uptake OR delay OR delayed OR delays OR time OR supply OR stigma OR support OR economics OR pathway OR pathways OR "side effect" OR "side effects" OR "adverse effect" OR "adverse effects" OR motivat*) | 15281159 |
| 4 | #1 AND #2 AND #3 | **3095** |
| 5 | TITLE-ABS-KEY ("semi-structured" OR semistructured OR unstructured OR informal OR "in-depth" OR indepth OR "face-to-face" OR structured OR interview* OR discussion OR discussions OR questionnaire OR questionnaires OR survey OR surveys OR surveyed OR "focus group" OR "focus groups" OR qualitative OR ethnography OR ethnographic OR fieldwork OR "field work" OR "key informant" OR randomized OR randomised OR randomization OR randomisation OR placebo OR randomly OR trial OR groups OR "clinical trial" OR "clinical trials" OR "evaluation study" OR "evaluation studies" OR "intervention study" OR "intervention studies" OR "case-control" OR cohort OR "longitudinal” OR longitudinally OR "prospective" OR prospectively OR "retrospective" OR "follow up" OR "comparative study") | 6282153 |
| 6 | #4 AND #5 | 1314 |
| 7 | TITLE-ABS-KEY (child OR children OR infant OR infants OR pediatric OR teenager OR teenagers OR teenaged OR teen OR teens OR "young adult" OR "young adults" OR adolescent OR adolescents OR youth) | 6618686 |
| 8 | #6 AND #7 | **839**  **as of 10.26.15** |

Africa Index Medicus

| Set # |  | Results |
| --- | --- | --- |
| 1 | “Tuberculosis” | 355 |
| 2 | “Africa” | 2875 |
| 3 | “Barrier” | 6 |
|  | #1 AND #2 AND #3 | **0**  **as of 10.26.15** |

Supplement 3. Cost barriers

| **Citation** | **Author** | **Year** | **Direct costs** | **Indirect costs** | **System costs** | **Carer costs** | **Costs prior to Diagnosis** | **Costs Diagnosis - Treatment** | **Level of analysis** | **Country** | **Synthesis** |
| --- | --- | --- | --- | --- | --- | --- | --- | --- | --- | --- | --- |
| 20 | Datiko, D. G. and B. Lindtjørn | 2010 | Y | Y | Y | N | N | N | Patient and system | Ethiopia | Community-based approaches are more cost-effective, than health facility-based approaches. Increased investment in community DOT and health extension workers can remove barriers of cost and distance and thus improve access to TB treatment in rural Ethiopia. |
| 19 | Vassall, A., A. Seme, P. Compernolle and F. Meheus | 2010 | Y | Y | N | Y | Y | Y | Patient | Ethiopia | TB and HIV patients incur major financial losses before and during treatment relative to income levels. For HIV infected patients with TB, delays were longer likely due to smear-negative TB, resulting in increased costs while patients pay for other ineffective treatments. Although TB and ART treatments are free, many patients still spend considerable money on diagnostic tests and drugs. Indirect costs are high during pre-treatment and patients do not turn to TB-HIV services first, leading to treatment delay. |
| 21 | Mauch, V., N. Woods, B. Kirubi, H. Kipruto, J. Sitienei and E. Klinkenberg | 2011 | Y | Y | N | Y | Y | Y | Patient | Kenya | There are substantial direct and indirect costs associated with TB with the majority of costs being indirect (i.e. inability to work). This increases poverty and leads to the 'medical poverty trap'. The Tool to Estimate Patients' Cost analysis is valuable and can be used to help identify interventions to lessen financial burdens on TB patients. It can also identify ways to reduce delays in diagnosis, decentralize services, and integrate TB/HIV care. |
| 22 | Umar, N. A., I. Abubakar, R. Fordham and M. Bachmann | 2012 | Y | N | N | N | Y | Y | Patient | Nigeria | Out of pocket spending is catastrophic with 9-39% of per capita GDP being spent on TB medications. This cost may result in delays, poor treatment outcomes, and continued spread of disease. |
| 23 | Laokri, S., M. Dramaix-Wilmet, F. Kassa, S. Anagonou and B. Dujardin | 2014 | Y | Y | N | N | N | N | Patient | Benin | Almost 75% of households in the survey experienced catastrophic expenditures with poorest of the poor having 36 times worse odds of experiencing catastrophic expenses. Lack of financial protection while using free TB services leads to inequities among individuals. |
| 24 | Yitayal, M., A. Aseffa, G. Andargie, L. Wassie and M. Abebe | 2014 | Y | Y | N | N | Y | Y | Patient | Ethiopia | Delays led to increased costs prior to diagnosis, which were much higher than post-diagnosis costs (2569 Birr versus 590 Birr). |
| 25 | Abimbola, S., K. N. Ukwaja, C. C. Onyedum, J. Negin, S. Jan and A. L. C. Martiniuk | 2015 | Y | N | N | N | Y | N | Patient | Nigeria | Referral linkages between formal and informal providers are important to increase early contact with appropriate providers, as transaction costs are a potential financial barrier of access to diagnosis and treatment of TB in Nigeria. Decentralization of services and community engagement may improve earlier contact with National TB Control Programs. |

Supplement 4. Health seeking behavior barriers

| Citation | Author | Year | Country | KAB | Care Pathway | Level of analysis | Synthesis |
| --- | --- | --- | --- | --- | --- | --- | --- |
| 52 | Salaniponi, F. M., A. D. Harries, H. T. Banda, C. Kang'ombe, N. Mphasa, A. Mwale, B. Upindi, T. E. Nyirenda, A. Banerjee and M. J. Boeree | 2000 | Malawi | Y | y | Patient | Fifty-seven percent (625) of participants thought they might have pulmonary TB prior to diagnosis. 70% of first contact was with professional care, 30% was with traditional healers, grocery shops, or local vendors. Decision of where to seek care was made solely by patient in 45% cases (494); influenced by a close family member in 24% (261); and healthcare worker in 20% (219) of cases. |
| 54 | Edginton, M. E., C. S. Sekatane and S. J. Goldstein | 2002 | South Africa | Y | Y | Patient | Thirty percent (91) of patients believed the cause of TB was unknown; 30% (90) percent believed TB was spread from others/inadequate previous treatment; 24% (71) of patients believed TB was caused by environmental/occupational exposure (e.g. mines); 11% (34) believed cigarette smoking/excess alcohol caused TB; and 6% (21) thought cultural reasons such as disobeying traditional rules or having sex transmitted TB. Thirty-five percent of patients (106) visited district hospitals first, 26% (77) visited traditional or faith healers first, 21% (64) visited district clinics, 10% (30) visited other hospitals, and 8% visited private doctors. *percentages rounded. |
| 53 | Enwuru, C. A., E. O. Idigbe, N. V. Ezeobi and A. F. Otegbeye | 2002 | Nigeria | Y | Y | Patient | Low level of knowledge and awareness of TB led to delays in diagnosis and treatment. Fifty to ninety-seven percent of patients had no knowledge of transmission or etiology of TB or knew the clinic(s) for effective diagnosis and treatment. Private and traditional medical providers are not trained in TB management and may have been a barrier/delay in appropriate management. |
| 55 | Eastwood, S. V. and P. C. Hill | 2004 | Gambia | Y | Y | Patient and system | There is little awareness of disease and knowledge of causes of TB, which creates barriers to treatment initiation. Duration of symptoms prior to presentation at medical facilities varied between four days and three years. Stigma from health care workers was associated with poverty, dirtiness, and prostitution. Many patients felt a lack of privacy in clinics, leading them to hide their diagnosis to avoid gossip. |
| 29 | Edginton, M. E., M. L. Wong, R. Phofa, D. Mahlaba and H. J. Hodkinson | 2005 | South Africa | Y | Y | Patient and system | Knowledge of TB was good in 63% of patients (657 interviews) although 51% did not know the cause of TB. Twenty-five percent of patients felt stigma was attached to TB diagnosis with AIDS as an association. Forty-one percent of patients attended three or more facilities prior to the TB hospital, and 5% had attended six facilities. |
| 28 | Yimer, S., G. Bjune and G. Alene | 2005 | Ethiopia | Y | Y | Patient and system | Patient delays are associated with first visit to a non-formal healthcare provider and self-treatment. Where to seek care was influenced by close family members and friends 89% of the time and health professionals 11% of the time. |
| 56 | Barker, R. D., F. J. C. Millard, J. Malatsi, L. Mkoana, T. Ngoatwana, S. Agarawal and S. De Valliere | 2006 | South Africa | N | Y | Patient and system | Fifty-one percent of patients (68) first visited a traditional healer, 17% (22) first visited a primary health center, 16% (21) visited a mixed traditional/Western medicine facility, 6% (8) visited a general practitioner, 5% (7) visited a faith healer, and 2% went directly to the hospital or it was not known. Patients who consulted traditional healers were more likely to die (31%) 24/77 than patients who went to a government health services (7%) 4/33 (p=0.004). Consulting a traditional healer was associated with death (OR 3.3 95%CI 1.05-10.38) although the effect was lost after adjusting for treatment delay. |
| 57 | Mfinanga, S. G., B. K. Mutayoba, A. Kahwa, G. Kimaro, R. Mtandu, E. Ngadaya, S. Egwaga and A. Y. Kitua | 2008 | Tanzania | Y | Y | Patient and system | Females had significantly more patient delays than males (p = 0.019). Risks for patient delay (not knowing that night sweats and chest pain are TB symptoms, believing that TB is always associated with HIV, being unemployed, and living far from a health facility were more than twice as high for females than males (OR = 2.22, 95% CI 1.14, 4.31). Nearly 37% (232) patients first sought care at dispensaries, 27.5% at district hospitals, 13.6% at health centers, and 13.4% at private facilities. |
| 58 | Dodor, E. A., S. Kelly and K. Neal | 2009 | Ghana | Y | N | Patient and system | There are five inter-related ways in which health professionals may stigmatize patients with TB: through isolation and exclusionary practices; behaviors of health professionals towards patients with TB; public health discourse; food safety and hygiene practices; and prohibition of full burial rites to those who died from TB. Legitimate precautions to prevent the spread of TB and stigmatizing attitudes and behaviors must be separated. |
| 51 | Yimer, S., C. Holm-Hansen, T. Yimaldu and G. Bjune | 2009 | Ethiopia | Y | Y | Patient and system | The median time for first health care action was 30 days (IQR 12-68). Being a dependent (for financial or physical needs) was significantly associated with not taking a health care action (AOR .39, 95%CI 0.2-0.7). Most participants did not receive timely diagnosis or treatment for their symptoms despite contacting a medical provider within a reasonable period of time after the onset of cough (30 days). Of participants who sought care (604), 30% (307) attended facilities that lacked diagnostic equipment. |
| 35 | Sendagire, I., M. Schim Van der Loeff, M. Mubiru, J. Konde-Lule and F. Cobelens | 2010 | Uganda | Y | Y | Patient and system | Patients who knew TB was curable were significantly less likely to have long total delay (AOR 0.28, CI 0.11-0.73) and patients who knew TB was curable were significantly less likely to have long patient delay (AOR 0.36, 95%ICI 0.13-.097). Of 242 patients 231 (95.5%) had seen at least one health care provider before the visit when their diagnosis of TB was made. Most providers the 169 patients visited were: private clinic of nurse or midwives (32.5%), drug shop (21.9%), and private doctor (18.3%). Nine percent already consulted the clinic and 8.3% consulted a hospital. |
| 59 | Dodor, E. A. | 2012 | Ghana | Y | Y | Patient | Patient delays in seeking TB treatment are often caused by lack of knowledge as many patients did not recognize symptoms of TB, and fear of stigma (from families, communities, and health professionals) as well as patient fears of TB itself. Many patients felt health workers' attitudes were demeaning which discouraged them from seeking care at the hospital. |
| 18 | Zimri, K., A. C. Hesseling, P. Godfrey-Faussett, H. S. Schaaf and J. A. Seddon | 2012 | South Africa | Y | N | Patient | Significant risk factors for non-attendance at TB clinics for children were Coloured ethnicity (OR 2.82, 95%CI 1.29-11.1, p=0.02), the mother being the TB source case (OR 3.78, 95%CI 1.29-11.1 p=0.02), and cigarettes smoked in the house (OR 2.37, 95%CI 1.01-5.57, p=0.04). Families who did not bring children to appointments were more concerned about risk of infection while waiting to be seen (OR 2.45, 95%CI 1.07-5.60, p=0.03) and felt like they had to wait a long time to be seen at local clinics (OR 2.47, 95%CI 1.07-5.69, p=0.03). When the mother is the source of TB infection, this can also be a barrier to care. Demographic, social, logistical, and cultural factors all contribute to the complexity of pediatric non-attendance at TB clinic appointments. |
| 36 | Ngangro, N. N., D. Ngarhounoum, M. N. Ngangro, N. Rangar, M. G. Siriwardana, V. H. des Fontaines and P. Chauvin | 2012 | Chad | Y | Y | Patient and system | Low economic status, low level of education, and belief in efficacy of traditional treatments were associated with extended diagnostic delay. In multivariate analysis, an extended patient delay was associated with low health score, an intermediate education level, misconceptions about TB treatment, and having no referral to a hospital. Thirty-three percent of patients sought treatment from a hospital, 22% by buying drugs on the informal market, 21% by visiting a health center, and 13% by using traditional medicine. Less than 8% of patients consulted a private doctor, and 3.5% consulted a pharmacist. Centralization of health care and/or its lack of quality may lead to delays due to the complex 'pathway of care' of referrals, diagnostic delays, and hierarchical levels of care. |
| 60 | Biya, O., S. Gidado, A. Abraham, N. Waziri, P. Nguku, P. Nsubuga, I. Suleman, A. Oyemakinde, A. Nasidi and K. Sabitu | 2014 | Nigeria | Y | Y | Patient | Patients with unsatisfactory knowledge, with multiple-care seeking, and with travel time > 20 minutes were more likely to delay seeking care from DOTS providers. After controlling for travel time and age, multiple care-seeking was independently associated with patient delay (AOR = 2.18, 95% CI = 1.09-4.35). Forty-one percent (66) of patients did not know TB was airborne and 27% (43) of TB patients first sought care from non-medical facilities. Involving traditional and non-medical providers in the referral chain of TB patients to DOTS centers is important to decrease delays. |
| 44 | Makwakwa, L., M. L. Sheu, C. Y. Chiang, S. L. Lin and P. W. Chang | 2014 | Malawi | Y | Y | Patient | Patients’ knowledge of TB was significantly associated with patient delay in both new (p=0.007) and retreatment cases (p=0.018). There were significant delays to initiation of TB treatment for both new and previously diagnosed patients, with most delays caused by the health system. The health system delay contributed more than 70% to the total delay. Retreatment cases with smear negative results had even longer health system delays. |
| 45 | Asefa, A. and W. Teshome | 2014 | Ethiopia | Y | N | Patient and system | Gender, perceived stigma, education status and family size significantly contribute to total delay. Being female, having tertiary education, and living in a larger family were less likely to have total delay. |
| 25 | Abimbola, S., K. N. Ukwaja, C. C. Onyedum, J. Negin, S. Jan and A. L. C. Martiniuk | 2015 | Nigeria | Y | Y | Patient | A 17.9% preference for alternative medicine and an 11.5% mistrust of the public sector were reasons patients sought care from care providers outside the public sector. Reducing transaction costs should include effective decentralization of services to integrate TB care with services at the primary health care level and community engagement to help keep informal providers within legal limits and facilitate referral linkages with formal and informal providers to increase early contact with appropriate TB care. |
| 61 | Cremers, A. L., M. M. de Laat, N. Kapata, R. Gerrets, K. Klipstein-Grobusch and M. P. Grobusch | 2015 | Zambia | Y | N | Patient and system | Eighty-two percent (113/138) patients with TB reported being stigmatized with females having more stigma (OR 5.479, 95%CI 1.51-19.88, p=.010). Stigma related to TB was associated with HIV, immoral behavior, incurability of disease, and traditional myths about TB etiology. Stigma led to patient hospital delays and poor treatment compliance and undermined efforts to screen for TB. |

Supplement 5. Infrastructure barriers

| Citation | Author | Year | Country | Geography | Laboratory Capacity | Level of Care | Initial loss to follow-up | Health Services Delay/Quality | Level of analysis | Synthesis |
| --- | --- | --- | --- | --- | --- | --- | --- | --- | --- | --- |
| 15 | Beyers, N., R. P. Gie, H. S. Schaaf, S. van Zyl, E. D. Nel, J. M. Talent and P. R. Donald | 1994 | South Africa | Y | N | N | N | Y | Patient and system | Nine percent (16) of children were not notified of TB diagnosis due to physician error. Of 156 notified with TB 26% (25) were not placed on treatment at local health clinics. Children in farming areas had the longest delays. |
| 26 | Lienhardt, C., J. Rowley, K. Manneh, G. Lahai, D. Needham, P. Milligan and K. P. McAdam | 2001 | Gambia | Y | N | Y | N | Y | System | The median number of providers seen prior to starting TB treatment was four (IQR 2–11). The majority of patients (73, 48.6%) were referred to a treatment center by a government nurse or a doctor. Health provider delays caused the majority of the total delay. Those living in rural areas also reported longer delay to treatment. Insufficient knowledge of TB symptoms among health providers of different types significantly contributes to provider delay. Increased awareness of TB among all types of providers and the general population (both urban and rural) is needed. |
| 27 | Martin, A., J. P. Baptiste and G. Krieger | 2004 | Chad | N | N | Y | N | Y | System | Significant treatment delays are caused by patients seeking care from non-medical providers or from "peripheral" hospitals. Total median delay was 75 days (min. 9 days, max. 790 days) with a median patient delay of 45 days (0-730 days) and median physician delay of 3 days (0-270 days). |
| 30 | Cambanis, A., M. A. Yassin, A. Ramsay, S. Bertel Squire, I. Arbide and L. E. Cuevas | 2005 | Ethiopia | Y | Y | N | N | Y | System | Causes for delay greater than four weeks included: rural residence, transport greater than two hours, overnight travel, and use of traditional medicine. Staying away for a night (e.g. for sputum investigation) added to out of pocket expenses. |
| 29 | Edginton, M. E., M. L. Wong, R. Phofa, D. Mahlaba and H. J. Hodkinson | 2005 | South Africa | Y | N | Y | N | Y | System | Forty-six percent (403) of patients reported directly to the hospital without referral, 29% were referred from a clinic, and 15% were referred from a private doctor. There was lack of documentation in hospital notification systems in addition to lack of specific referrals being made by name. Almost half (219 of 443) of patients attending clinics expected problems with continuing TB treatment due to clinic access (106), long wait times (78), and clinic staff problems (35). |
| 28 | Yimer, S., G. Bjune and G. Alene | 2005 | Ethiopia | Y | N | Y | N | Y | Patient and system | Living greater than 10km from a health facility was significantly associated with an increased patient delay (OR =0.42, 95%CI .24, .72). Patients seeking health care from a pharmacy or unregulated retailer or clinician (rather than medical provider) had a four-fold increase in delay. The median health-seeking period was 15 days (IQR 15-21) and health providers' delay was 61 days (IQR 31-116). |
| 31 | Dembele, S. M., H. Z. Ouedraogo, A. I. Combary, B. Sondo, J. Macq and B. Dujardin | 2006 | Burkino Faso | Y | Y | Y | N | Y | System | The case-finding rate was 19.5% which was similar to the national level; and inadequate compared to the 70% WHO target. Case-finding rates varied by health center and did not only depend on patients' geography/health seeking behavior. |
| 16 | Engelbrecht, A. L., B. J. Marais, P. R. Donald and H. S. Schaaf | 2006 | South Africa | N | Y | N | N | Y | System | In the majority of cases (55%) treatment was either not given or initiated without knowledge of the culture result. Delay between admission and clinical diagnosis was a median of 1 day (range 0-21) and treatment was started simultaneously. For children with a culture diagnosis, delay was a median of 40 days (range 16-68). Culture diagnosis may be important as clinical diagnosis, especially in the presence of HIV may be difficult to make. However, the majority of cultures add little diagnostic value to children with TB; although DST can only be completed if a culture is available and more drug resistance testing is needed. |
| 32 | Botha, E., S. den Boon, K. A. Lawrence, H. Reuter, S. Verver, C. J. Lombard, C. Dye, D. A. Enarson and N. Beyers | 2008 | South Africa | N | N | Y | Y | Y | System | Only 82% (2037) of the 2484 TB suspects had at least two sputum samples recorded and 18% of TB suspects had deficiencies in the collection process, receiving, and recording of results. Initial defaulters (range 0-40%) with fixed PHC having higher rates (18%) versus mobile PHC (14%). |
| 33 | Botha, E., S. Den Boon, S. Verver, R. Dunbar, K. A. Lawrence, M. Bosman, D. A. Enarson, I. Toms and N. Beyers | 2008 | South Africa | N | N | N | Y | Y | System | Twenty-four percent (14/58) of initial defaulters died, of the remaining 44 patients, 26 could not be found, 18 were interviewed. Fifty-six percent of the reasons for initial default were directly linked to services. |
| 34 | den Boon, S., S. Verver, C. J. Lombard, E. D. Bateman, E. M. Irusen, D. A. Enarson, M. W. Borgdorff and N. Beyers | 2008 | South Africa | N | N | N | Y | Y | System | Of the 20 actively detected cases who started treatment, 16 (80%) were successfully treated, which was a similar proportion as in passively detected cases (OR 1.01, 95 % CI 0.33–3.09). Seven percent (2/27) of the actively detected TB case died, compared to 4% (18/473) of passively detected TB cases, however, this difference was not significant (OR 2.02, 95%CI 0.44–9.20). |
| 20 | Datiko, D. G. and B. Lindtjørn | 2010 | Ethiopia | Y | N | Y | N | N | System | Community based DOT involving HEWs cost less than health facility DOTS by 62.6%. In community DOT, 74.8% (172/230) of the patients were cured and 14.3% (33/230) completed treatment. Of the 88 patients treated in Health facilities DOTS, 68.2% (60) were cured and 15.9% (14) completed treatment. The incremental cost-effectiveness ratio of health facility DOT to community DOT was -16.3. Increased investment in Community DOT and HEW's can remove barriers of cost and distance, thus improving access to TB treatment in rural Ethiopia. |
|  | Sendagire, I., M. Schim Van der Loeff, M. Mubiru, J. Konde-Lule and F. Cobelens | 2010 | Uganda | Y | N | N | N | Y | Patient and system | Over 90% (231) of patients consulted more than one provider before diagnosis of TB was made with a median of four visits per patient (range 1-30). Less than 5% of TB suspects were diagnosed during their first visit to a healthcare provider. |
| 36 | Ngangro, N. N., D. Ngarhounoum, M. N. Ngangro, N. Rangar, M. G. Siriwardana, V. H. des Fontaines and P. Chauvin | 2012 | Chad | N | N | Y | N | Y | Patient and system | Thirty-three percent (95) patients sought treatment from a hospital, 22% (63) by buying drugs on informal market, 21% (60) by visiting a health center, 13% (37) by using traditional medicine, less than 8% (23) by consulting a private doctor, and 3.5% (10) by consulting a pharmacist. Two percent (6) did not seek care. Health system delays were 2.4 times longer than patient delays *No confidence intervals given. |
| 37 | Scott, V., V. Azevedo and J. Caldwell | 2012 | South Africa | N | Y | Y | N | Y | System | Rapid cycle evaluation is important for improving program evaluation and to decrease treatment commencement time. Practical steps to take and includes how to integrate services, especially HIV and pediatric services. |
| 17 | Seddon, J. A., A. C. Hesseling, M. Willemse, P. R. Donald and H. S. Schaaf | 2012 | South Africa | N | N | N | N | Y | Patient and system | Median time to treatment initiation 58 days (IQR 25-120) was shorter when there was a known adult MDR-TB index case than when no adult index case was known (median time 123 days, IQR 67-231, p<.001). Despite advanced disease and extra pulmonary disease in more than 30% of children, 80% had favorable outcomes with 12% (13) overall mortality rate regardless of treatment initiation. |
| 41 | Cowan, J., J. G. Cowan, S. Barnhart, S. Demamu, D. Fiseha, W. Graham, E. Melese, L. Reason, F. T. Asfaw, G. Feleke and B. Feleke | 2013 | Ethiopia | N | N | Y | N | Y | System | Healthcare providers are aware of high success of DOTS but there is no system for follow-up with patients who default. Pharmacists had concerns for shortages of first-line TB medications and lack of pediatric dosages. Many participants reported inadequate prevention of disease transmission and worried about contracting TB themselves and discussed high rates of staff turnover. Many participants were frustrated at the inability to diagnose and treat MDR-TB. |
| 38 | Ebonwu, J. I., K. S. Tint and C. Ihekweazu | 2013 | South Africa | N | N | Y | Y | Y | Patient and system | Only 63% (593) of patients initiated treatment. Of the 37% (349) of patients who did not initiate treatment 84% (293) had previously been treated for TB. Patients referred from a hospital were eight times more likely not to initiate treatment than patients referred from a clinic (aOR 8.2, 95%CI 1.8-37.8). |
| 40 | Jacobson, K. R., D. Theron, E. A. Kendall, M. F. Franke, M. Barnard, P. D. van Helden, T. C. Victor, E. M. Streicher, M. B. Murray and R. M. Warren | 2013 | South Africa | N | Y | N | N | Y | System | MTBDRplus, significantly reduced time from specimen collection to initiation of MDR TB treatment from a median of 80 days to 55 days for patients in a rural Western Cape, South African hospital. The laboratory phase decreased delay from a median 55 days (IRQ 46-66) for culture based testing to median of 27 days (IRQ 20-34) when using MTBDRplus. Smear-positive cases took a median of 22 days (IRQ 13-26) to process before resistance was reported compared with a median 29.5 days (IRQ 24-38.5) for smear-negative. Transport for samples remained a median of two days for both DST groups. |
| 39 | Yassin, M. A., D. G. Datiko, O. Tulloch, P. Markos, M. Aschalew, E. B. Shargie, M. H. Dangisso, R. Komatsu, S. Sahu, L. Blok, L. E. Cuevas and S. Theobald | 2013 | Ethiopia | Y | Y | Y | Y | Y | System | HEWs increased case notifications from 64 to 127/100,000 population/year. Distance and accessibility were barriers for patients, especially women, poor, elderly, and the very sick. Lab technicians provided additional training to HEW when smears were of poor quality through QI projects. 100% of patients with TB diagnosed in the intervention zone initiated treatment during the implementation period. |
| 43 | Ansa, G. A., J. D. Walley, K. Siddiqi and X. Wei | 2014 | Ghana | N | N | Y | N | Y | System | The doctor-led approach to ART becomes a barrier to integrating with the decentralized, nurse-led approach to TB treatment. Increasing service integration improved HIV screening but not co-trimoxazole preventive therapy or antiretroviral therapy across three hospitals. There was insufficient data to identify the most effective model of service from the study. |
| 45 | Asefa, A. and W. Teshome | 2014 | Ethiopia | Y | N | N | N | Y | Patient and system | The median patient delay was 30 days (IQR 20.2-60), diagnostic delay 7 (IQR 3-14), treatment delay 3 (IQR 1-4) and total delays measured in days were 45 days (IQR 34.5-69.5). Forty-nine percent (150/306) of patients did not initiate treatment within 45 days of symptom onset. |
| 42 | Dlamini-Mvelase, N. R., L. Werner, R. Phili, L. P. Cele and K. P. Mlisana | 2014 | South Africa | N | N | N | Y | Y | System | Thirty-six percent (98) of patients with confirmed samples were not found on the MDR-TB treatment register within three months of Xpert samples. Of patients initiating treatment, 28% (75) commenced treatment within two weeks, 40% (107) within one month, 21% (56) within two months, and 8% (21) within three months. Three percent (8) of patients never initiated treatment. |
| 44 | Makwakwa, L., M. L. Sheu, C. Y. Chiang, S. L. Lin and P. W. Chang | 2014 | Malawi | Y | Y | N | N | Y | System | There were significant delays to TB treatment initiation for both new and previously diagnosed patients, with most delays caused by the health system. Retreatment cases with smear negative results had even longer health system delays. |
| 46 | Virenfeldt, J., F. Rudolf, C. Camara, A. Furtado, V. Gomes, P. Aaby, E. Petersen and C. Wejse | 2014 | Ginea-Bissau | N | N | N | N | Y | Patient and system | The median delay to treatment decreased during the study period - in the first year median delay was 14.6 weeks (IQR 9.3-26.1) and dropped to 8.6 weeks (IQR 5.7-16.7) in the last year. In linear regression, delay decreased by 10.3% (7.9-12.6%) annually. |
| 47 | Yimer, S. A., G. A. Bjune and C. Holm-Hansen | 2014 | Ethiopia | Y | Y | Y | N | Y | Patient and system | Patients from rural areas had a three-fold increase in patients’ delay compared to those from urban areas (aOR 3.4; 95%CI 1.3-8.9). Extra-pulmonary TB more likely to experience delay in seeking treatment compared to PTB (aOR 2.6; 95%CI 1.3-5.4). Improved TB diagnostic and treatment facilities in rural areas could reduce diagnostic and treatment delays. |
| 49 | Cox, H. S., J. F. Daniels, O. Muller, M. P. Nicol, V. Cox, G. van Cutsem, S. Moyo, V. De Azevedo and J. Hughes | 2015 | South Africa | N | Y | Y | N | Y | System | Decentralization and introduction of LPA and Xpert testing reduced time from sputum sample collection to date of second-line anti-TB treatment initiation from a median of 71 days (IQR 49-134, n=158) in 2003-2006 to a median of 8 days (IQR 5-25, n=89) in 2013 (p <.0001). |
| 50 | Ross, J. M., A. Cattamanchi, C. R. Miller, A. J. Tatem, A. Katamba, P. Haguma, M. A. Handley and J. L. Davis | 2015 | Uganda | Y | N | Y | N | N | System | Patients referred for TB evaluation travelled further to care centers than those without referrals for TB; however, longer distance or travel time was not a barrier to evaluation and treatment initiation. Neither distance nor travel time predicted completion of TB evaluation. |
| 48 | Van Den Handel, T., K. H. Hampton, I. Sanne, W. Stevens, R. Crous and A. Van Rie | 2015 | South Africa | Y | Y | Y | Y | Y | System | Decentralization coupled with Xpert testing had the shortest time to treatment initiation. Xpert increased the proportion of cases with bacteriological confirmation, however, point-of-care treatment may resulted in fewer people being evaluated for TB. |
